# Dendrimer-Based Coatings on a Photonic Crystal Surface for Ultra-Sensitive Small Molecule Detection

**DOI:** 10.3390/polym15122607

**Published:** 2023-06-08

**Authors:** Ruslan Shakurov, Svetlana Sizova, Stepan Dudik, Anna Serkina, Mark Bazhutov, Viktorija Stanaityte, Petr Tulyagin, Valery Konopsky, Elena Alieva, Sergey Sekatskii, Julia Bespyatykh, Dmitry Basmanov

**Affiliations:** 1 Lopukhin Federal Research and Clinical Center of Physical Chemical Medicine of Federal Medical Biological Agency, 1A Malaya Pirogovskaya Street, 119435 Moscow, Russia; sv.sizova@gmail.com (S.S.); stepan.dudik@rcpcm.org (S.D.); anna.serkina@gmail.com (A.S.); mark.bazhutov@rcpcm.org (M.B.); stanaytite_v_a@student.sechenov.ru (V.S.); juliabespyatykh@gmail.com (J.B.); basmanov.dmitry@gmail.com (D.B.); 2 Research Institute for Systems Biology and Medicine (RISBM), Nauchniy Proezd 18, 117246 Moscow, Russia; tylagin@vivaldi.net; 3 Shemyakin & Ovchinnikov Institute of Bioorganic Chemistry RAS, 16/10 Miklukho-Maklaya Street, 117997 Moscow, Russia; 4 Institute of Spectroscopy RAS, 5 Fizicheskaya Street, Troitsk, 108840 Moscow, Russia; konopsky@gmail.com (V.K.); alieva@isan.troitsk.ru (E.A.); 5 Laboratory of Biological Electron Microscopy, Institute of Physics (IPHYS), BSP 419, Ecole Polytechnique Fédérale de Lausanne, and Department of Fundamental Biology, Faculty of Biology and Medicine, University of Lausanne, CH1015 Lausanne, Switzerland; serguei.sekatski@epfl.ch; 6 Expertise Department in Anti-Doping and Drug Control, Mendeleev University of Chemical Technology of Russia, 9, Miusskaya Square, 125047 Moscow, Russia; 7 Institute of Physics and Technology, 9 Institutskiy Pereulok, 141701 Dolgoprudny, Russia

**Keywords:** photonic crystal surface mode biosensor, PC SM, biosensor coating, hyperbranched polymers, dendrimer, PAMAM, microfluidics, label-free detection, oligonucleotides, biomolecular interaction

## Abstract

We propose and demonstrate dendrimer-based coatings for a sensitive biochip surface that enhance the high-performance sorption of small molecules (i.e., biomolecules with low molecular weights) and the sensitivity of a label-free, real-time photonic crystal surface mode (PC SM) biosensor. Biomolecule sorption is detected by measuring changes in the parameters of optical modes on the surface of a photonic crystal (PC). We describe the step-by-step biochip fabrication process. Using oligonucleotides as small molecules and PC SM visualization in a microfluidic mode, we show that the PAMAM (poly-amidoamine)-modified chip’s sorption efficiency is almost 14 times higher than that of the planar aminosilane layer and 5 times higher than the 3D epoxy-dextran matrix. The results obtained demonstrate a promising direction for further development of the dendrimer-based PC SM sensor method as an advanced label-free microfluidic tool for detecting biomolecule interactions. Current label-free methods for small biomolecule detection, such as surface plasmon resonance (SPR), have a detection limit down to pM. In this work, we achieved for a PC SM biosensor a Limit of Quantitation of up to 70 fM, which is comparable with the best label-using methods without their inherent disadvantages, such as changes in molecular activity caused by labeling.

## 1. Introduction

The label-free approach, as the name suggests, implies the detection of bioligand analytes without reporter molecules. Different optical label-free sensor techniques to detect bioligands have been developed, such as the quartz crystal microbalance (QCM) [1,2], plasmon resonance (SPR) [3,4,5], localized surface plasmon resonance (LSPR) [6], resonant waveguide grating (RWG) [7,8], resonant mirror (RM) [9,10], high-Q optical microcavities [11,12], and two-dimensional materials-based photodetectors [13,14,15,16,17]. The sensitivity of these methods (i.e., the signal-to-noise ratio) decreases with the size of the molecules immobilized on the biosensor sensitive layer. To date, the optical detection limit for small molecules, i.e., low-molecular-weight molecules, is in the range from μM to nM [18] and in the range of up to pM for SPR-based methods [19].

One more label-free technique is high-precision multiparameter biomarker detection using the PC SM method [20,21,22,23,24,25]. PC is a material characterized by the periodic modulation of refractive indices to the scale of the light wavelength, which allows for the long-range propagation of surface optical waves along the outer surface of a biochip sensitive layer. One of the ways of increasing biosensor sensitivity is to modify the sensitive layer. We recently developed and published an approach of making 3D coatings based on dextrans with various anchor groups (aldehyde, carboxy, epoxy) and different molecular weights suitable for binding bioligands for the high-performance sorption of small biomolecules and their subsequent detection with a PC SM biosensor in real time [26].

The detection parameter of bioligand label-free binding analysis—the signal-to-noise ratio—is extremely low due to both the small number of bound molecules and their small size [27]. Obviously, there are two ways to solve this problem: signal amplification and noise reduction [27]. Signal amplification can be achieved by increasing the density of biomolecules immobilized on a chip surface by means of a branched 3D structure formation on the sensitive layer. A large number of surface modification methods are known, such as silanes treatment [28,29,30], cationic polyelectrolytes [31,32], low-molecular-weight linkers [33], avidin-functionalized coatings [34], self-assembled monolayers [35,36], and polysaccharides with anchor active groups [26,37,38].

PAMAM (poly-amidoamine) is a class of dendrimer that is made of repetitively branched subunits of amide and amine functionalities. Since their discovery [39,40], PAMAMs have attracted attention due to their unique properties. Unlike traditional polymers, PAMAM dendrimers are polyvalent, practically monodisperse structures with a polydispersity index of 1.01–1.05 whose size and surface functional group number can be precisely controlled during synthesis.

Due to their monodispersed properties, PAMAM layers deposited as modifying coatings have well-reproducible characteristics in contrast to materials with similar functions, such as nanoparticles or cationic polymers [41].

PAMAMs have found their application in the pharmaceutical and biomedical fields [41]. However, there are currently limited data about the physicochemical properties of surfaces modified with PAMAMs, which retain their flexible structure after covalent binding to a surface, as well as about the utilization of such modified surfaces in biomolecular sensors [42,43,44].

One more characteristic property of PAMAM is multivalency [45,46]. It has been shown that multivalency can lead to a synergistic effect of enhancing affinity, i.e., the activity increase is more than can be expected from mechanically summing the valence capabilities of a system’s functional groups—this effect is usually called the ”dendritic” effect [47]. It is important to distinguish between this “dendritic” effect and the additive effect of a simple increase in binding efficiency, where there are several moles of binding molecules per mole of ligand [48]. Due to their spherical shape and high density of terminal reactive groups, PAMAMs seem to be promising materials for biochip sensitive layer modification. Modification can significantly increase the amount of bioligands immobilized on a chip surface and improve sensitivity by “signal amplification”.

PAMAMs are used as drug and gene delivery systems [49,50,51] and antimicrobial [52,53] and antiamyloid agents [54]. The biomedical applications of dendrimers are based on their ability to bind biomolecules through terminal active groups, or the “guest–host” mechanism [41]. PAMAM dendrimers have been shown to be useful for the design of optical sensors. Most of the studies have demonstrated proof-of-concept for detecting pH, heavy metals, and other analytes; PAMAMs are additionally used as composite parts for making quantum dots, metal particles, etc. [55]. There are a few reports about PAMAM-based SPR sensors [5,56], but there are no reports on the use of PAMAMs as surface modifiers for PC SM biosensors.

## 2. Materials and Methods

### 2.1. Materials

PAMAM dendrimers with ethylenediamine cores of generation 4.0 at 10 wt. % in methanol (stock solution, PAMAM), 99% (3-Aminopropyl)triethoxysilane (APTES), glutaraldehyde (GA), bovine serum albumin (BSA, heat shock fraction, pH 7, ≥98%), sulfo-NHS-biotin, streptavidin (STP, from Streptomyces avidinii, 97%), and phosphate-buffered saline (PBS) were obtained from Sigma-Aldrich (St. Louis, MO, USA). Other chemicals were of analytical grade and used without additional purification. Milli-Q water (Millipore, Merck KGaA, Darmstadt, Germany) of the highest purification with a resistivity of 18 MΩ cm was used to prepare all the solutions.

All oligonucleotides (Table 1) were synthesized and purified with Evrogen (Moscow, Russia) and solubilized in Milli-Q water.

### 2.2. Photonic Crystal Surface Mode Detection System

Registration of the biomolecule interaction process on the surface was performed in real time using an “EVA 2.0” microfluidic label-free PC SW biosensor (PCbiosensors.com accessed on 22 April 2023, Russia) [21,22,26]. The sensitive surface was a final layer of silicon oxide in a one-dimensional photonic crystal (1D PC). The following 1D PC structure was designed using the impedance approach [57,58] and was used in the experiments: (BK-7-substrate)/H (LH)^2^ L′/(water), where L is a SiO_2_ layer with a thickness of d1 = 215.8 nm, H is a TiO_2_ layer with d2 = 70 nm, and L′ is a SiO_2_ layer with d3 = 369.7 nm. The SiO_2_/TiO_2_ 6-layer structure (started from TiO_2_ and finished by the SiO_2_ layers) was produced through ion-assisted e-beam deposition.

The optical scheme of the EVA 2.0 biosensor was based on angle interrogation of a PC SM. A stabilized laser beam detected both the PC surface mode (by s-polarization) and the critical angle total internal reflection (by p-polarization). The optical surface mode resonance angular interrogation measured the thickness of the adsorbed layer, and a simultaneous detection of the critical angle total internal reflection provided independent data on the liquid’s refractive index.

### 2.3. Fabrication of the PC Biochip

#### 2.3.1. PC Activation and Functionalization

Before activation and functionalization, PC chips were thoroughly cleaned of organic contaminants according to [26]. The PC chips were submersed in Hellmanex detergent (Hellma, Muellheim, Germany), ultrasonicated with double-distilled water and ethanol three times for 10 min, and dried under nitrogen flow. Next, the cleaned PC chips were treated in a Zepto W6 plasma cleaner (13.56 MHz/100 W, Diener Electronic, Ebhausen, Germany) for 10 min at 600–800 mbar air pressure to create silanol and siloxane groups on the sensitive PC layer.

After cleaning, the PCs were immersed in APTES solution (3%, *v*/*v*) for 3 min, rinsed with double-distilled water, and baked for 30 min at 120 °C to remove the moisture (dehydration) present on the PC surface. Quality control of the silanization process was carried out using atomic force microscopy (AFM) (see Section 2.5).

#### 2.3.2. Functionalization of the PC chip

**Figure 1:**
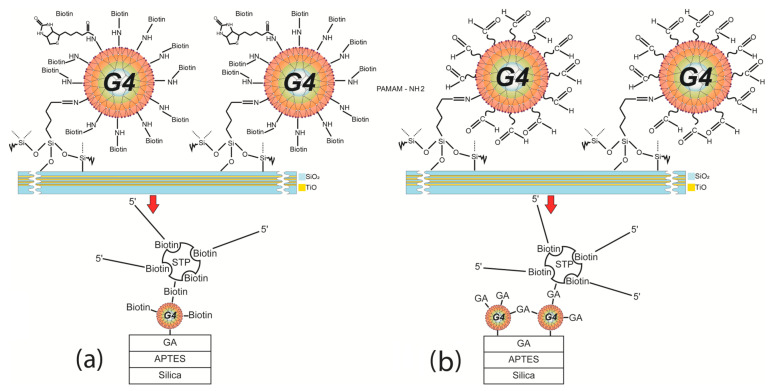
Schematic illustration of (**a**) poly-amidoamine dendrimers of generation 4.0 (PAMAM G4)-GA, and (**b**) PAMAM G4-biotin functionalization of the photonic crystal (PC) chip.

After the cleaning and activation (silanization) procedure, the PC chips were immediately functionalized with PAMAMs (Figure 1). We used 4.0-generation PAMAMs in all the experiments (PAMAM G4).

##### Functionalization with PAMAM G4-GA (First Method)

Activated PC chips were treated with a 0.1% solution of GA in water for 10 min, rinsed with water to remove excess GA, and dried under nitrogen flow. After that, PAMAM G4 1% solution in PBS at pH 7.2 was applied on the GA-treated PC chips for 10 min, rinsed with PBS at pH 7.2, treated again with GA (molar ratio of PAMAM G4:GA was 1:32) for 5 min, and rinsed with water. Prepared PAMAM G4-GA PC chips were kept in PBS at pH 7.2 until used in experiments.

##### Functionalization with Biotinylated PAMAM G4 (Second Method)

Biotinylated PAMAM G4 (PAMAM G4-biotin) was synthesized as reported in [59] with minor modifications. PAMAM G4 in 100 µL of stock solution was dissolved in 100 mM sodium bicarbonate buffer (pH 9.0) to a mass concentration of 0.1%, and NHS-biotin was added to create molar ratios of 1:8 and 1:64. The mixture was stirred for 24 h at RT and then dialyzed against water to remove unconjugated biotin for 12 h at 4 °C.

PC chip modification with PAMAM G4-biotin was carried out in a microfluidic flow cell of the PC SM biosensor. GA (0.1% solution in PBS, pH 7.2), followed by PAMAM G4-biotin in PBS at pH 7.2, was run through a flow cell of the bio sensor at a flow rate of 50 μL/min until the signal plateaued (app. 10 min for each stage). The system was thoroughly rinsed with PBS solution after each stage.

### 2.4. Protein and Low-Molecular-Weight Biomolecule Binding Detection

#### 2.4.1. Protein Detection

The model protein BSA in a 0.1 mg/mL solution in PBS at pH 7.2 was used to test binding detection with the PAMAM-G4-GA-modified PC chip surface. BSA solution was run through a flow cell until the signal plateaued, and then PBS was run through to remove unconjugated BSA. BSA binding with the PC chips was monitored in situ with a PC SM biosensor.

Detection of 0.05 mg/mL STP in PBS at pH 7.2 with the PAMAM-GA-modified PC chip surface was carried out in the same way as BSA detection.

#### 2.4.2. Low-Molecular-Weight Biomolecule Detection

Some oligonucleotides previously designed for *Mycobacterium tuberculosis* spacer oligonucleotide typing (spoligotyping) were used to test the ability of the PC SM biosensor to detect small molecules.

Thus, in the Z36-S36 pair, the Z36 oligonucleotide (oligosensor) was the sequence for detecting the model ssDNA target of S36 (oligotarget), and in the Z37-S37 pair, the Z37 oligonucleotide was the sequence for detecting the model ssDNA target S37 sequence. The probe sequences of the oligosensor were biotinylated, which enabled specific binding to the sensitive surface of the PC chip. All oligonucleotides were used at a concentration of 25 pM/mL in PBS at pH 7.2.

STP (0.05 mg/mL in PBS) was incubated with a biotinylated oligosensor for 10 min at RT for complex preparation (STP-biotinylated oligosensor). STP (0.05 mg/mL in PBS), a STP-biotinylated oligosensor, a BSA blocking agent (0.1 mg/mL in PBS), and a biotinylated oligosensor or oligotarget were consecutively run through a microfluidic flow cell with one of the PAMAM-G4-modified PC chips at a flow rate of 50 μL/min until the signal plateaued, at which point the system was thoroughly rinsed with PBS solution.

### 2.5. Atomic Force Microscopy

All images were obtained with an AFM NTEGRA PRIMA I (NT-MDT, Moscow, Russia), and CS37 B cantilevers (NT-MDT, Russia) were used. The cantilevers had a 0.1 N/m force constant, a 16 kHz resonant frequency in an air medium, and an 8 nm tip radius. The semicontact mode scanning method was used, and AFM studies were conducted in a liquid medium (Milli-Q water) to prevent PAMAM damage and deformation upon drying. Substrate modal tilt was removed with Fit Line X filters. AFM measurements were performed directly on a PC.

## 3. Results

The method based on the label-free optical detection of biomolecule interactions using a photonic crystal surface mode (PC SW) biosensor allows for real-time tracking of molecule interactions, meaning conclusions about the interaction affinity can be drawn [22]. The previously proposed approach based on creating a 3D dextran matrix on a 1D photonic crystal (PC) surface allowed us to achieve a 20% increase in sorption capacity [26]. Nevertheless, the transition to multiplex detection based on the imaging mode of PC biosensing [20] requires a much larger sorption capacity to detect small molecules, i.e., up to the pM range.

In this study, a poly-amidoamine dendrimer of generation 4.0 (PAMAM G4) was chosen to create a 3D matrix on a PC surface. Using glutaraldehyde (GA) as a crosslinking agent, individual dendrimer molecules were bound to the PC chip surface and conjugated into complex structures with each other, as confirmed by the results of atomic force microscopy (AFM) in Section 3.5. Thus, the measured height of the dendrimer molecule was 4.5 nm [60], and the average layer thickness was 70 nm, with individual conjugates reaching up to 120 nm (see Section 3.5).

### 3.1. Model Protein-Binding Capacity on the PAMAM G4-GA PC Chip

The PC surface was activated, and (3-Aminopropyl)triethoxysilane (APTES) was applied to create a layer with terminal amino groups (see Section 2.3.1). The next step was GA treatment, which could proceed via two routes: (1) involving two dialdehyde carbonyl groups and two adjacent silane amino groups in the formation of Schiff bases or (2) involving one silane amino group and one glutaraldehyde carbonyl group, with the second amine group remaining free and reactive. The second mechanism provided an option to further modify the PC surface using PAMAM G4 with terminal amino groups.

The use of PAMAM G4 and GA at a molar ratio of 1:32 PAMAM G4:GA led both to inter-dendrimer interaction with the formation of imine bonds and to the appearance of terminal carbonyl groups capable of further interaction.

In order to evaluate the specific binding of the model protein to the PC chip’s PAMAM-G4-functionalized surface, we studied the change in the PC-based biosensor’s surface layer thickness at successive stages of its surface pretreatment and in a model experiment. Bovine serum albumin (BSA) was used as a model protein to detect binding to the PAMAM G4-GA layer of the PC chip. Experiments with BSA were carried out in PBS at pH 7.2. BSA solution (0.1 mg/mL) was run through the flow cell at a rate of 50 μL/min until signal stabilization and then rinsed with PBS for 2 min.

**Figure 2:**
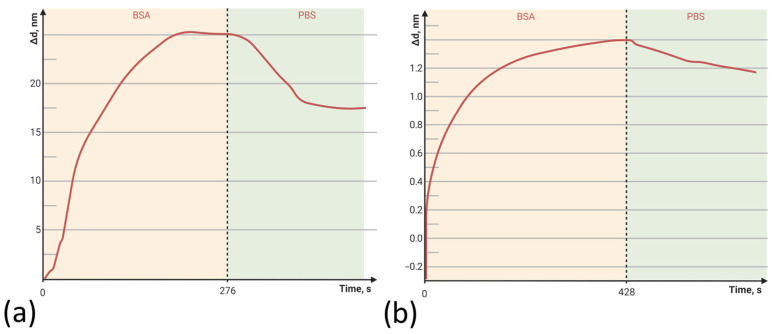
The change in adlayer thickness upon binding of bovine serum albumin (BSA) with the (**a**) PAMAM-G4-GA-modified and (**b**) (3-Aminopropyl)triethoxysilane (APTES)-modified PC surfaces.

Figure 2a,b show the sensorgrams (change in the adlayer thickness as a function of time) of BSA binding to the PC surface functionalized with PAMAM G4-GA (outer PC surface). For comparison, we used a method of modifying the PC chip surface with APTES [26]. The data obtained show a 14-fold increase in the sorption capacity of the PC sensitive layer modified with PAMAM G4-GA compared to the APTES-modified PC (Table 2).

### 3.2. Streptavidin Binding Capacity on the PAMAM G4-GA PC Chip

Next, we evaluated the specific binding of STP to the PAMAM-G4-functionalized surface of the PC chip. STP was the key component of the small molecule detection complex. Experiments with STP (0.05 mg/mL) were carried out in the same way as with BSA (Section 3.1).

The fabrication of a 3D PAMAM-G4-GA-based structure on the PC chip surface made it possible to significantly increase the sorption capacity of biomolecules. In the case of protein detection, such as with STP or avidin, the 64 terminal amino groups in the structure of PAMAM G4 made it possible both to increase the sorption capacity of the PC surface and to increase the sorption specificity by covalently binding a biotin molecule to each PAMAM G4 terminal amino group.

**Figure 3:**
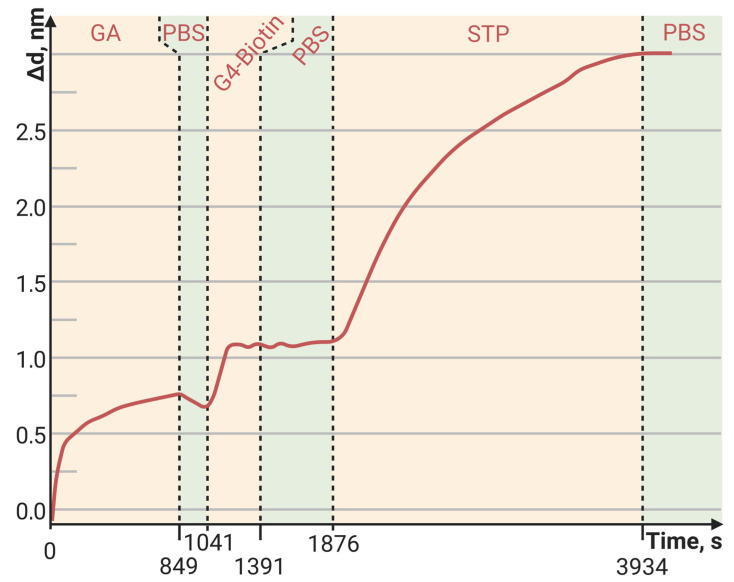
The change in adlayer thickness upon binding of STP to the PAMAM-G4-biotin (molar ratio of 1:8)-modified PC surface.

**Figure 4:**
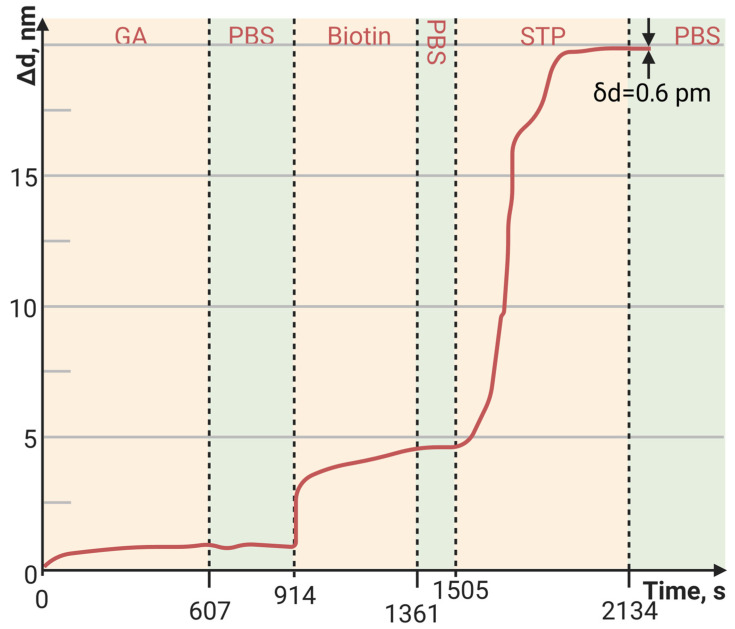
The change in adlayer thickness upon binding of STP to the PAMAM-G4-biotin (molar ratio of 1:64)-modified PC surface.

The sorption capacity of PAMAM G4-biotin prepared at different molar ratios was also investigated. The sensorgrams of STP bound to the PAMAM-G4-biotin-modified PC surface at molar ratios 1:8 and 1:64 of PAMAM G4:biotin are shown in Figure 3 and Figure 4, respectively.

**Figure 5:**
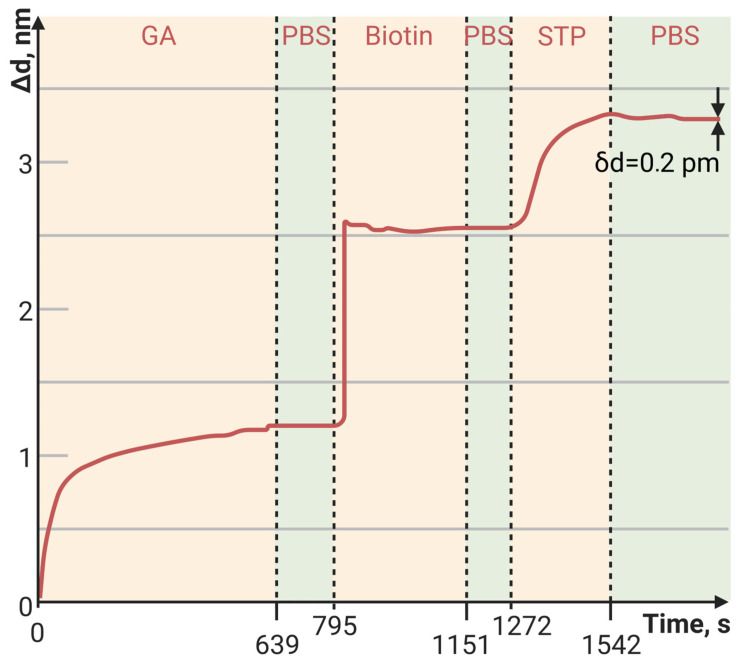
The change in adlayer thickness upon binding of STP to the biotin-modified PC surface.

Here, we see that an eight-fold increase in the biotinylating degree of PAMAM G4 resulted in an almost eight-fold increase in the amount of bound STP, i.e., the increase was completely specific. Both the minimum and maximum biotinylating degrees of PAMAM G4 provided a greater amount of bound protein than the planar PC surface modified with biotin (Figure 5, Table 3).

Thus, we determined that the method for PC surface modification that provided the maximum sorption of proteins (on the examples of BSA and STP) was PAMAM G4-biotin with a molar ratio of 1:64.

### 3.3. Detection of Low-Molecular-Weight Biomolecule Interaction with PC SM Biosensor

The main task was to evaluate the possibility of detecting small biomolecule sorption and interaction using a PC SM biosensor. For this, STP-biotinylated oligosensor complexes (STP-biotinylated Z36, STP-biotinylated Z37) were prepared and bound to the surface of a PAMAM-G4-biotin-modified PC chip.

The detection capacity of oligonucleotide sequence interaction was carried out with the PAMAM-G4-biotin (molar ratio of 1:64)-modified PC chip. A complex STP-biotinylated oligosensor was prepared and bound to the PAMAM G4-biotin PC chip. For comparison, we used PCs modified with APTES and with PAMAM G4-GA.

**Figure 6:**
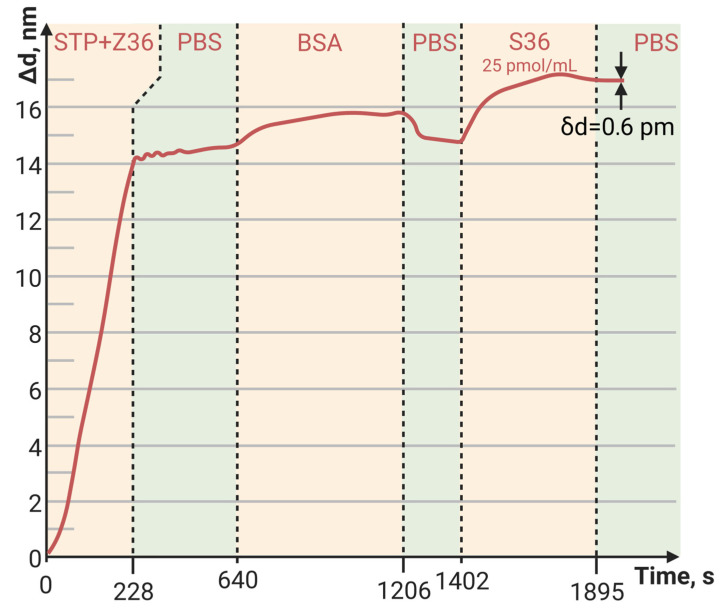
The change in adlayer thickness upon binding of the STP-biotinylated oligosensor Z36 complex and the following complementary oligotarget of S36 to the PAMAM-G4-GA-modified PC surface.

**Figure 7:**
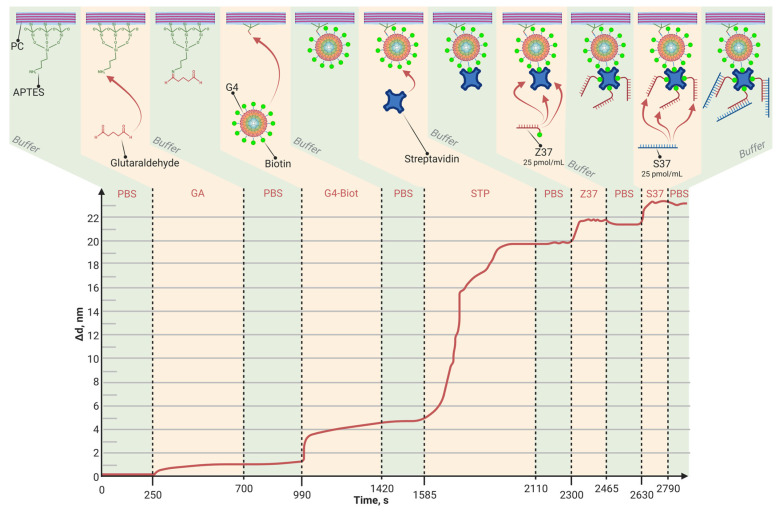
The change in adlayer thickness upon binding of the STP-biotinylated oligosensor Z37 and the following complementary oligotarget of S37 with the PAMAM-G4-biotin-modified PC surface.

**Figure 8:**
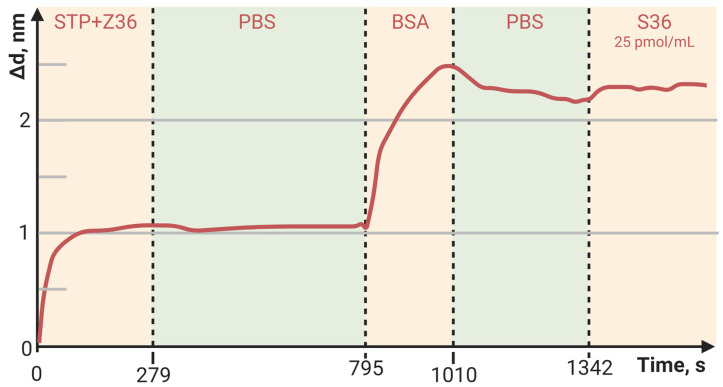
The change in adlayer thickness upon binding of the STP-biotinylated oligosensor Z36 complex and the following complementary oligotarget of S36 to the APTES-modified PC surface.

Figure 6, Figure 7 and Figure 8 show the sorption curves (sensorgrams) of the oligosensor–STP complex—the oligosensor and its complementary oligotarget on the PC surface—modified with PAMAM G4-GA, PAMAM G4-biotin, and APTES, respectively. Comparative data are given in Table 4. The greatest oligotarget increase was obtained for the PAMAM-G4-GA-modified surface and could be explained by nonspecific binding to the PAMAM-G4-GA-modified PC surface.

### 3.4. Sensor Baseline Noise, LoD, LoQ, and Dynamic Range

The baseline noise, or standard deviation of the baseline thickness (STD (d_a_)), for the current biosensor was δd = 2 × 10^−4^ nm for thin, tight adlayers (such as the biotin-modified PC surface in Figure 5), while the baseline noise for thick, loose adlayers (such as in Figure 4 and Figure 6) was 6 × 10^−4^ nm. To estimate the sensitivity of the biosensor, the latter STD was used. We defined the Limit of Detection (LoD) as 3 × the standard deviation of the baseline and the Limit of Quantitation (LoQ) as 10 × the standard deviation of the baseline. In Figure 4, the injection of a streptavidin concentration of 0.05 mg/mL led to a thickness increase of 15 nm (see Table 3), resulting in the S/N ratio of 25,000 (15 nm/0.0006 nm). Therefore, in this case, the LoQ for streptavidin in the PAMAM G4-biotin layer was 20 pg/mL (0.05 [mg/mL]/2500). On the other hand, in Figure 6, the concentration of oligonucleotides at 25 pM/mL resulted in a thickness increase of 2.1 nm (see Table 4), giving the S/N ratio of 3500 (2.1 nm/0.0006 nm). The LoQ for oligonucleotides in the PAMAM G4-GA layer was 70 fM/mL (25 [pM/mL]/350).

The dynamic range (DR) of the current biosensor was limited by the size of the sensor’s matrix. The 25 nm shift in Figure 4 amounted to ~14% of the DR, indicating that the overall operating range of the biosensor in terms of adsorption thickness was approximately 180 nm.

### 3.5. Atomic Force Microscopy

**Figure 9:**
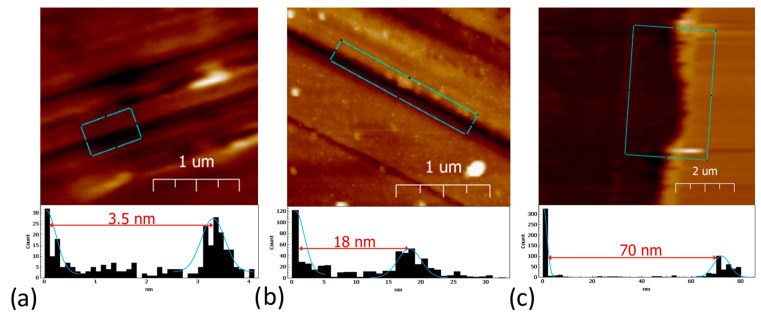
AFM topology image and height histogram: (**a**) APTES-modified PC surface, (**b**) APTES-PAMAM G4-biotin layer, (**c**) APTES-GA-PAMAM G4 layer.

Atomic force microscopy (AFM) was chosen as an independent method for surface investigation at various stages of PC chip formation and biomolecule detection. To control the quality of the APTES-treated PC sensitive surface (see Section 2.3.1), we created a scratch and measured the thickness of the APTES coating as we described earlier [26]. AFM studies were conducted in a liquid medium (Milli-Q water) to prevent the PAMAM G4 layer from damage and deformation upon drying. Figure 9 illustrates the topology of the scratches made on different PCs modified with APTES, APTES-PAMAM G4-biotin, and APTES-GA-PAMAM G4. The scanning area was 10 × 10 um, and the number of points was 256 × 256. The scan rate was 1 Hz. Height histograms allowed us to measure coating thickness [32]. As seen in the histogram in Figure 9a, the mean thickness of the APTES layer was 3.5 nm, and the mean height of roughness was 0.5 nm. In agreement with the data given in [61], we assume that the silanization protocol chosen to modify the PC chip made it possible to obtain uniform coatings with a reproducible surface topology.

The mean height of the PAMAM G4-biotin coating was 18 nm, and the mean height of roughness was 5.3 nm (Figure 9b). Results for the PAMAM-GA layer on the modified PC surface are shown in Figure 9c. The mean thickness was 70 nm, and the mean height of roughness was 9.5 nm. The results obtained are consistent with the data from [56,57]. Authors have shown that generations of PAMAMs with lower molecular weights have smaller heights [62,63]. Therefore, generation 4.0 of PAMAM should have molecules of about 10 nm in height, which means that our data agree with these works.

## 4. Discussion

The PC surface modification approach proposed in this article significantly increased PC chip sorption capacity. In general, we could say that, in comparison to a silane-modified planar PC surface, the response was 8–10 times greater both for relatively large biomolecules, such as proteins, and relatively small biomolecules, such as oligonucleotides. This work is a continuation of a previous paper published in this journal [26]. The thickness of PAMAM-based coatings measured using AFM (18 and 70 nm) was comparable to that of 3D ED-matrix-based coatings (range of 10–40 nm), but the values of sorption capacity compared to previously developed 3D polysaccharide coatings on PC surfaces increased by 5–6 times.

The choice of dendrimer generation was based on known [44] considerations that structural flexibility is important for the formation of dendrimer complexes with DNA molecules, the reduction of which occurs at higher (five and more) generations of PAMAMs. The assumption of increased sorption capacity at higher dendrimer generations due to a much greater number of terminal amino groups was rejected on the basis that steric considerations do not allow all capable groups to participate in binding. This, however, requires further investigation.

Here, we proposed two approaches to PC surface modification. The first approach consisted of the creation of a 3D matrix of PAMAM G4 globules crosslinked with GA on a PC sensitive surface. However, despite all efforts, this matrix formation approach had a stochastic character, as confirmed with AFM by the presence of aperiodic large clusters on the PC.

The second approach, based on the preliminary specific biotinylation of PAMAM G4 terminal amino groups, proved to be a reliable and reproducible method for fabricating effective sensor biochips.

The achieved response values (Δd = 1.5–2 nm) during oligonucleotide detection on the sensorgrams gave us the possibility to detect concentrations of an order of magnitude lower than those used. In combination with the achieved detection time (3–5 min), this allowed us to classify the method as ultra-sensitive and fast.

## 5. Conclusions

In this work, we proposed approaches for PC sensitive surface functionalization with dendrimers. We developed and studied PAMAM-G4-based PC coatings. The resulting 3D structures on the PC surface provided an increase in sorption capacity of over 10 times compared to that of the PC planar surface, as well as more than 5–6 times compared to 3D structures based on dextran with different anchor groups. The proposed methods of PC surface modification showed their effectiveness both for large molecules, such as proteins, and for small molecules, such as oligonucleotides.

## Figures and Tables

**Table 1:** Oligonucleotide sequences used in the experiments.

Name	Sequence 5′→3′	Length, Nt	Mw, Da
S36, oligotarget	TTCGGGAGCATGCCGCAGCTGCGGATGTGGTGCTGGATTTCGA	43	13,321
Z36, oligosensor	CGAAATCCAGCACCACATCCGCAGCTG-(Biotin)	27	8157
S37, oligotarget	CTGAAAGGGGGACTGTGGACGAGTTCGCGCTCAAAAT	37	11,467
Z37, oligosensor	GAGCGCGAACTCGTCCACAGTCCC-(Biotin)	24	7261

**Table 2:** The adlayer thickness (Δd, nm) of the PAMAM-G4-GA-modified and APTES-modified PC surfaces.

Outer PC Surface	APTES	PAMAM G4-GA
Δd, nm	1.2	16.5

**Table 3:** Comparative data of STP adlayer thickness (Δd, nm) bound to the PC sensitive layer modified with biotin and PAMAM G4-biotin at different molar ratios.

Outer PC Surface	Biotin	PAMAM G4-Biotin (1:8)	PAMAM G4-Biotin (1:64)
Δd, nm	0.8	1.9	15

**Table 4:** Comparative data of oligotarget adlayer thickness bound to PC sensitive layers modified with APTES, PAMAM G4-GA, and PAMAM G4-biotin.

Outer PC Surface	APTES	PAMAM G4 GA	PAMAM G4-Biotin
Δd, nm	0.11	2.1	1.7

## Data Availability

Not applicable.
